# Conversion of a light-driven proton pump into a light-gated ion channel

**DOI:** 10.1038/srep16450

**Published:** 2015-11-24

**Authors:** A. Vogt, Y. Guo, S. P. Tsunoda, S. Kateriya, M. Elstner, P. Hegemann

**Affiliations:** 1 Institute of Biology, Experimental Biophysics, Humboldt-Universität zu Berlin, 10115 Berlin, Germany; 2 Institute of Physical Chemistry, Karlsruhe Institute of Technology, 76131 Karlsruhe, Germany; 3 Department of Biochemistry, University of Delhi South Campus, New Delhi, India

## Abstract

Interest in microbial rhodopsins with ion pumping activity has been revitalized in the context of optogenetics, where light-driven ion pumps are used for cell hyperpolarization and voltage sensing. We identified an opsin-encoding gene (CsR) in the genome of the arctic alga *Coccomyxa subellipsoidea* C-169 that can produce large photocurrents in *Xenopus* oocytes. We used this property to analyze the function of individual residues in proton pumping. Modification of the highly conserved proton shuttling residue R83 or its interaction partner Y57 strongly reduced pumping power. Moreover, this mutation converted CsR at moderate electrochemical load into an operational proton channel with inward or outward rectification depending on the amino acid substitution. Together with molecular dynamics simulations, these data demonstrate that CsR-R83 and its interacting partner Y57 in conjunction with water molecules forms a proton shuttle that blocks passive proton flux during the dark-state but promotes proton movement uphill upon illumination.

Microbial rhodopsins, which are widely distributed across archaea, bacteria, and some eukarya branches, are categorized as sensory rhodopsins, ion channels, and ion pumps^1^,^2^. They are light-activated, seven-transmembrane proteins with an all-*trans* retinal as a chromophore. Photon absorption redistributes electrons within the protonated retinal Schiff base that promotes isomerization around the 13-*cis* bond followed by a cyclic cascade of proton transfer, water redistribution, and conformational changes within the protein that may be monitored by UV/Vis and infrared spectroscopy^3^,^4^,^5^. In the context of optogenetics, outward directed proton pumps, as well as inward directed chloride pumps, can be utilized as neural silencers or fluorescence-based voltage sensors^6^,^7^,^8^.

The function of all microbial proton pumps within the native environment or when applied experimentally depends absolutely on their performance at moderate to high electrochemical load. Surprisingly, our knowledge about the contribution of individual residues to proton pump strength remains very limited despite extensive research on bacteriorhodopsin (BR) since 1971. These studies have mostly been conducted by spectroscopic methods using BR in native purple membranes or BR mutants in detergent at zero voltage. Only the voltage dependence of a few pumps has been determined by electrical measurements of *Xenopus laevis* oocytes; however, the photocurrent amplitudes in these experiments were too small for more comprehensive studies^9^,^10^,^11^,^12^,^13^. Therefore, various conflicting issues regarding the transport mechanism of proton pumps remained unresolved, including the origin of the proton driving force, the background for voltage dependence of the photocycle, and the functional importance of the non-conserved proton release group.

Here we report our characterization of *Coccomyxa* Rhodopsin (abbreviated as CsR), a proton-pumping rhodopsin from the unicellular arctic freshwater alga *Coccomyxa subellipsoidea* C-169. This psychrotolerant alga resides in temperatures ranging from −50 °C to +25 °C^14^. CsR produces huge photocurrents in oocytes as well as in HEK-293 cells, which will enable researchers to study the molecular transport steps in greater detail than BR has ever allowed. We capitalized on this advantage by characterizing CsR-WT and various mutants. This examination identified the highly conserved CsR-R83 and its interaction partner CsR-Y57 (R82 and Y57 in BR) as the central positions for maintaining directionality and full pump strength. We demonstrate that CsRs with mutations in these residues alone exhibit low active transport forces, if any, and operate as passive light-gated proton channels already at low electrochemical load. Such light-activated proton selective channels will be of great interest for optogenetic applications in which a specific proton release is required (e.g., in lysozymes or neuronal vesicles).

## Results

### Proton transport model for CsR

**Figure 1:**
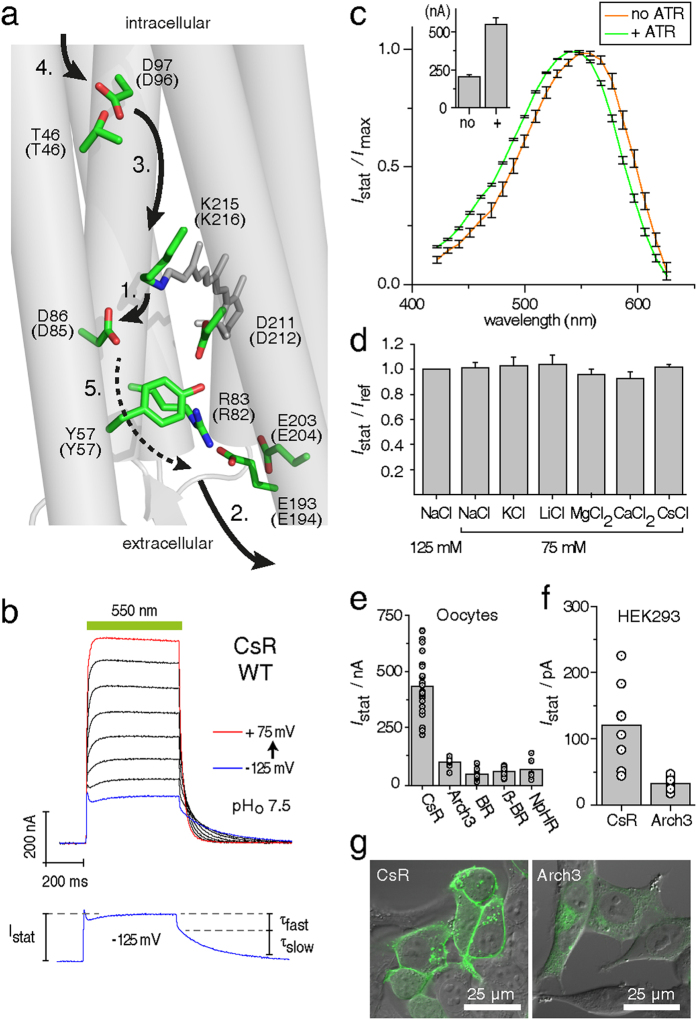
Basic characterization of CsR in *Xenopus laevis* oocytes and HEK-293 cells. (**a**) Proposed structure of CsR-WT with mutated residues based on the BR crystal structure (PDB: 1M0L). Brackets indicate respective positions in BR and demonstrate the high conservation of key residues. Arrows highlight the proposed proton transport pathway of CsR-WT, which is similar to BR. A dotted arrow is used to indicate that whether R83 deprotonates itself during the photocycle remains to be determined. (**b**) Typical photocurrents of CsR-WT measured in oocytes at extracellular pH_o_ 7.5 after illumination with 550 ± 25 nm. Holding potentials were increased from −125 mV in 25 mV steps up to +75 mV. Separated current trace illustrates the biexponential decay after light illumination. (**c**) Action spectra measured in oocytes at pH 7.2 with supplementation of 5 μM all-*trans* retinal (ATR, n = 7) and without ATR (n = 5). Currents were normalized to the maximal stationary currents. Inset shows absolute amplitudes at 0 mV (ATR, n = 16; without ATR, n = 21). (**d**) Ion selectivity measured in oocytes at pH_o_ 7.2 and 0 mV (all n = 5 except CsCl, n = 3). (**e**) Absolute photocurrent amplitudes of diverse microbial pumps at pH_o_ 7.5 as measured in oocytes (all at 0 mV, illumination with 550 ± 25 nm, CsR [n = 24], Arch3 [n = 8], BR [n = 19], βHK-BR [n = 17], NpHR [n = 6]). (**f**) Absolute photocurrent amplitudes of CsR-WT-EGFP (n = 8) and Arch3-WT-EGFP (n = 11) measured in HEK-293 cells (0 mV, 550 ± 7 nm, pH_o_ 7.2, pH_i_ 7.2 after 24–30 h, 1 μM ATR). (**g**) Confocal images of HEK-293 cells transfected with CsR-WT-EGFP or Arch3-WT-EGFP (after 24–30 h, 1 μM ATR). Scale bars represent 25 μm. Data represent the mean ± SE.

The amino acid sequence of CsR is 37% homologous to BR (Supplementary Fig. 1), with the regions important for transport highly conserved between the two proteins (Fig. 1a). Thus, a transport mechanism for CsR can be derived based on that generally accepted for BR^15^. Briefly, all-*trans* retinal is linked to K215 of the protein (K216 in BR) via a protonated retinal Schiff-base (RSBH^+^), which is stabilized by a negatively charged counterion complex consisting of D86 and D211 (D85/D212 in BR). After photo-isomerization of RSBH^+^ and partial rotation of the N-H dipole, the pK_a_ of RSBH^+^ is dramatically decreased and the proton is transferred to D86 (D85 in BR, L to M transition)^16^. D86 protonation causes R83 (R82 in BR) to switch outward towards the proton release group residues E193 and E203 (E194 and E204 in BR), resulting in proton release into the extracellular medium^15^,^17^. In the second part of the photocycle, the deprotonated Schiff-base becomes accessible from the cytoplasmic site by forming a transient water chain between the retinal Schiff-base and the proton donor CsR-D97 (D96 in BR)^15^,^18^. The pK of D97 in darkness is high (>11 in BR) due to hydrogen bridging to T46 (Fig. 1a)^19^. Conformational changes of the protein disrupt this bond, thereby lowering the pK of D97 and allowing a proton to transit along the novel water chain to reprotonate the RSB (M to N transition). Next, CsR-D97 is reprotonated from the cytoplasmic bulk phase during the N to O transition. At the end of the photocycle, during the transition from O-state to the dark-state, the proton is transferred from D86 to the proton release group, passing or bypassing R83 and Y57 (Fig. 1a). Although the exact mechanism of this transfer has yet to be elucidated, transient protonation of D211 may be involved^20^.

### Photocurrents of CsR-WT

CsR-expressing oocytes supplemented with all-*trans* retinal were illuminated for 0.5 seconds with green light of wavelength 550 ± 25 nm at holding voltages ranging from −125 mV to +75 mV (Fig. 1b). CsR exhibited active proton pumping, and photocurrents were always directed outward and graded with the membrane voltage. At negative voltages, a minor initial transient current was observed. Darkness resulted in bi-exponential decay as reported earlier for BR and *Acetabularia* rhodopsin^12^,^21^. The fast component was less voltage-dependent (τ_fast_ = 4 ± 1 ms), which is in contrast to the slow component that accelerated from negative (τ_slow_ = 260 ± 15 ms at −125 mV) to positive voltage (τ_slow_ = 28 ± 2 ms at +75 mV). This characteristic resembles the O- to dark-state transition used for voltage sensing with Arch3 in the neuroscience field^8^. Wavelength variation revealed the sensitivity maximum at 545 nm (Fig. 1c). Without supplementation of all-*trans* retinal, amplitudes were reduced and the action spectrum maximum was red-shifted by 10 nm, most likely due to binding of endogenous (3,4)-didehydro-retinal^22^,^23^. Exchange of extracellular cations or replacement of chloride for other anions did not affect photocurrent amplitudes or kinetics supporting the exclusive proton transport (Fig. 1d). Most surprisingly, in all conditions examined, the photocurrent amplitudes were much larger than those generated by Arch3-, NpHR-, BR-, or β-BR^21^ expressing oocytes (Fig. 1e). Expression and membrane targeting of CsR in HEK-293 cells was brilliant and superior to Arch3, resulting in larger photocurrents (Fig. 1f,g). This observation provided us with the opportunity to analyze the functionality of key residues in CsR with unrivaled precision.

### Channel-like photocurrents in CsR mutants

#### Active site mutants

**Figure 2:**
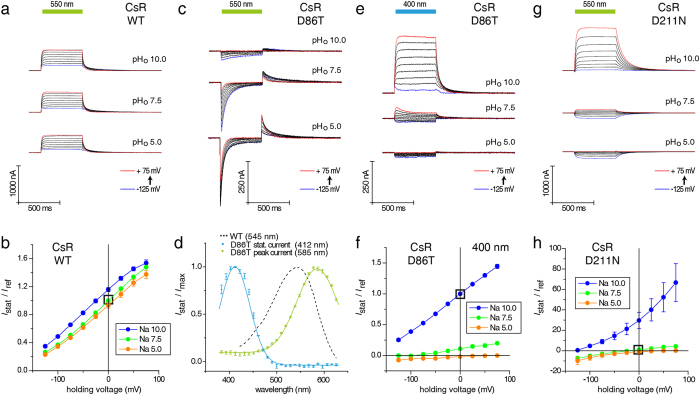
Current traces and *I*_Stat_(*E*) of CsR-WT, CsR-D86T, and CsR-D211N at different extracellular pH values. (**a**) Photocurrents of CsR-WT were less dependent on pH and always outward directed (550 ± 25 nm). (**b**) *I*_Stat_(*E*) of stationary photocurrents of CsR-WT (550 ± 25 nm, normalized to pH 7.5 and 0 mV, pH 10 [n = 10], pH 7.5 [n = 25], pH 5 [n = 7]). (**c**) Photocurrents of CsR-D86T upon illumination with green light (550 ± 25 nm). Inward-directed peak currents were observed, especially at low pH_o_. These are followed by outward directed peaks immediately after illumination. (**d**) Action spectra show two separated protein populations of CsR-D86T. Both are shifted towards CsR-WT as shown by dotted lines. Spectrum of stationary currents was measured at pH 10 and 0 mV (n = 4). Spectrum of peak currents in green was measured at pH 7.5 and 0 mV (n = 4). (**e**) Stationary photocurrent traces of CsR-D86T upon illumination with blue light (400 ± 25 nm). Stationary currents disappear at low pH. (**f**) *I*_Stat_(*E*) of stationary currents of CsR-D86T (400 ± 25 nm, normalized to pH 10 and 0 mV, pH 10 [n = 13], pH 7.5 [n = 8], pH 5 [n = 5]). (**g**) Photocurrents of CsR-D211N are directed outward and inward at high and low pH_o_, respectively (550 ± 25 nm). (**h**) *I*_Stat_(*E*) of stationary photocurrents of CsR-D211N (550 ± 25 nm, normalized to pH 7.5 and 0 mV, pH 10 [n = 6], pH 7.5 [n = 7], pH 5 [n = 6]). Data were measured in oocytes and represent the mean ± SE.

WT and mutant CsRs were compared at different extracellular pH values (pH_o_) and holding voltages to determine the contribution of specific residues to active proton pumping. As expected, photocurrents of CsR-WT showed low dependence on the extracellular pH (Fig. 2a,b). Replacement of the counterion D86 with T, as found in the chloride pump halorhodopsin, blocked the stationary current completely. At pH 5 and 7.5, CsR-D86T exhibited transient currents of reduced size that were directed inward, suggesting a unique inward charge transfer (Fig. 2c). This transient current can be explained as deprotonation of RSBH^+^ during the photocycle with an intramolecular proton transfer towards the intracellular bulk phase. In all proton-pumping rhodopsins, D85 or its homologs stabilize the high pK of the Schiff base nitrogen during all-*trans* configuration of the dark-state^2^. In CsR-D86T, the RSBH^+^ was destabilized and the missing charge of D86 was only partially substituted by the secondary more distant charged residue D211, as visualized by the absorption shift from 545 to 585 nm (Fig. 2d). The concomitant drop in the RSB-pK caused deprotonation at pH_o_ 10 and eliminated the transient photocurrents. Instead, 400 nm light evoked stable and stationary outward currents (Fig. 2e,f). At pH_o_ 7.5 and 5.0, the fraction of CsR with deprotonated Schiff Base was small but still measurable. Moreover, the reversal voltage shifted to more positive values as expected for a passive transport. These data demonstrate that CsR-D86T with deprotonated RSB operates as a light-gated proton conductor with residual pump activity. Illumination with bright white light completely abolished residual pumping and shifted the reversal voltages to the Nernst values (Supplementary Fig. 2a). Similar currents were observed previously when the corresponding Proteorhodopsin (PR-D97T) mutant was expressed in oocytes^13^. For PR-D97T blue light-induced stationary currents were recorded only at pH_o_ 7.4 and −30 mv, suggesting inward pumping without passive proton conductance. A similar phenomenon was observed for BR-D85T in experiments using black lipid membranes, but its voltage dependence was not studied^24^,^25^. These stationary currents were interpreted as chloride pumping. However, our data demonstrate that CsR-D86T is not involved in chloride pumping since substitution of chloride with gluconate did not affect the current size or current-voltage relationship (Supplementary Fig. 2b). Chloride conductance was also excluded from PR-D97T in the previous PR-study^13^.

Replacement of the second counterion residue D211 with N also converted active ion pumping into passive ion transport with variable vectoriality at neutral pH_o_, small inward currents at low pH_o_, and large outward currents at high pH_o_ (Fig. 2g,h). In this case, no residual pump activity was observed between pH_o_ 6 and 10 (Supplementary Fig. 2a). Moreover, the RSB remained protonated under all conditions, suggesting that proton network rearrangements, not the RSB protonation state, are responsible for decoupling the pump and the observed passive proton flow.

### Mutants of the proton release pathway

**Figure 3:**
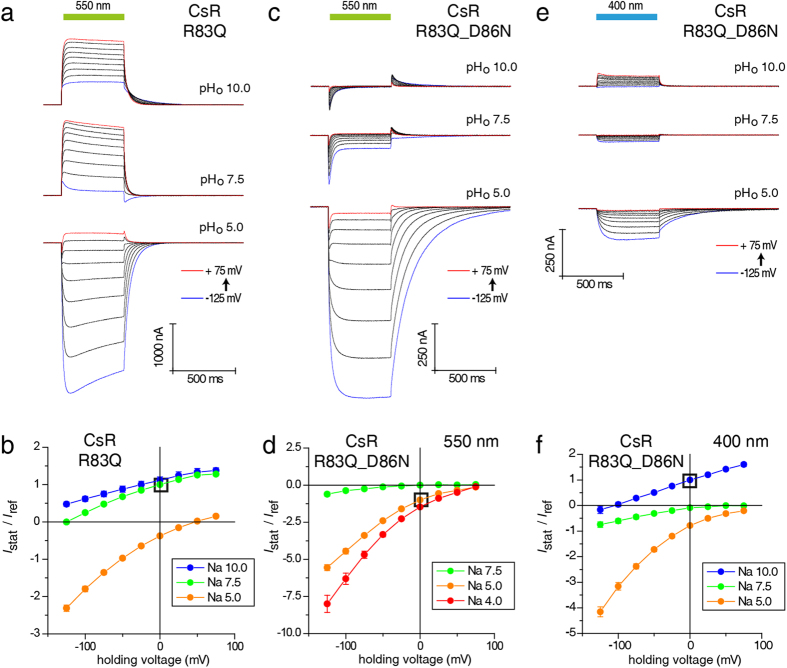
Current traces and *I*_Stat_(*E*) of CsR-R83Q and CsR-R83Q-D86N at different extracellular pH values. (**a**) Photocurrents of CsR-R83Q are characterized by strong inward-directed photocurrents at low pH_o_ (550 ± 25 nm). (**b**) *I*_Stat_(*E*) of stationary photocurrents of CsR-R83Q (550 ± 25 nm, normalized to pH 7.5 and 0 mV, pH 10 [n = 7], pH 7.5 [n = 10], pH 5 [n = 6]). (**c**) Photocurrents of CsR-R83Q-D86N (550 ± 25 nm). (**d**) *I*_Stat_(*E*) of CsR-R83Q-D86N stationary photocurrents (550 ± 25 nm, normalized to pH 5 and 0 mV, pH 7.5 [n = 7], pH 5 [n = 9], pH 4 [n = 5]). Double mutant shows that inward-directed channel-like currents are independent of the primary proton donor D86. (**e**) Stationary photocurrent traces of CsR-R83Q-D86N upon illumination of blue light (400 ± 25 nm). Similar to D86N, low outward-directed stationary currents were observed at high pH_o_. (**f**) *I*_Stat_(*E*) of CsR-R83Q-D86N stationary photocurrents (400 ± 25 nm, normalized to pH 10 and 0 mV, pH 10 [n = 5], pH 7.5 [n = 4], pH 5 [n = 5]). Data were measured in oocytes and represent the mean ± SE.

The proton release pathway connects RSBH^+^ (active site) with the extracellular surface. The main elements involved are the proton shuttle R83, the Y57 residue connecting R83 with D211, water molecules, and the proton release complex E193 and E203 (Fig. 1a). We investigated each of these critical components in CsR. Based on the assumed central role of R83 in the proton release pathway, we examined the effect of mutating this residue to Q. CsR-R83Q-mediated outward currents were larger than WT under standard conditions of pH_o_ 7.5 and pH_o_ 10 (Fig. 3a,b and Supplementary Fig. 3a). It as has been claimed that in the complementary mutant R82Q of BR the pK of D85 is raised from 2.7 to 7.2^26^. Owing to the action spectra of the CsR-R83Q that are similar at pH = 5 and pH = 10 and both spectra are 5–10 nm blue shifted compared to WT (Supplementary Fig. 3d,e), deprotonation of the D86 in CsR is unlikely. At pH_o_ 5, the currents generated by R83Q were directed inward with amplitudes twice as large as the outward currents observed at pH_o_ 7.5. However, the current decay and photocycle kinetics were slower. These data suggest collapsed pump activity in favor of an inward rectifying passive conductance. The *I(E)* diagram indicates that some residual pump activity remained because the reversal potentials for all three pH_o_ values, especially for pH_o_ 7.5, were more negative than expected for a purely passive channel (Fig. 3b, Supplementary Fig. 3c). In the R83Q-D86N double mutant, active pumping was disrupted completely while the passive inward current remained fully intact. These experiments demonstrate again that passive proton influx is independent of proton transfer from RSBH to the acceptor residue D86 (Fig. 3c,d). Light at 400 nm only promotes small stationary inward and outward currents (Fig. 3e,f).

**Figure 4:**
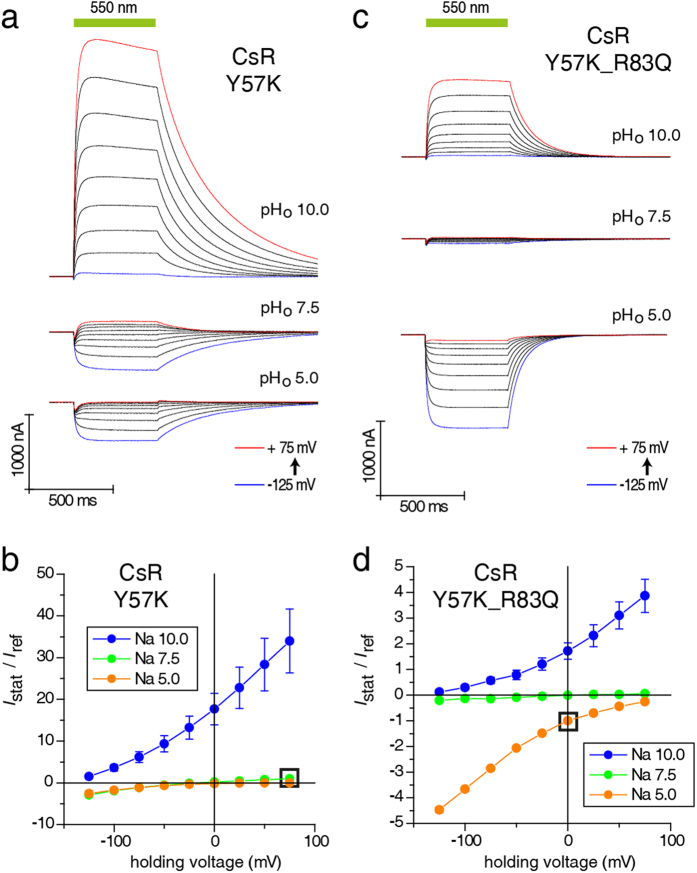
Current traces and *I*_Stat_(*E*) of CsR- Y57K and CsR-Y57K-R83Q at different extracellular pH values. (**a**) Photocurrents of CsR-Y57K are characterized by strong outward-directed photocurrents at high pH_o_ (550 ± 25 nm). (**b**) *I*_Stat_(*E*) of stationary photocurrents of CsR-Y57K (550 ± 25 nm, normalized to pH 7.5 and +75 mV, pH 10 [n = 6], pH 7.5 [n = 9], pH 5 [n = 4]). (**c**) Photocurrents of CsR-Y57K-R83Q exhibit the properties of both single mutations but without huge amplitudes at high electrochemical loads (550 ± 25 nm). (**d**) ***I***_**Stat**_(***E***) of stationary photocurrents of CsR-Y57K (550 ± 25 nm, normalized to pH 5 and 0 mV, pH 10 [n = 9)], pH 7.5 [n = 9], pH 5 [n = 9]). Data were measured in oocytes and represent the mean ± SE.

Y57 connects the counterion residue D211 to R83 via a single water molecule^27^ (Fig. 1a). To examine the role of Y57 in CsR, we mutated it into K because channelrhodopsins contain lysine at this location (e.g., K132 in C1C2)^28^. CsR-Y57K was an outward-directed ion channel that produced small passive inward currents at low pH_o_ and large outward currents at high pH_o_ with slower kinetics (τ_off_ = 318 ± 25 ms at pH 10 and +75 mV, Fig. 4a,b). The current amplitudes generated by both CsR-R83Q and CsR-Y57K were larger than those of CsR-WT (Supplementary Fig. 3a), suggesting that the number of transported protons per photocycle varied. Interestingly, the CsR-Y57F mutant was still able to pump with moderate power in a pH-dependent manner (Supplementary Fig. 3b). The pump activity of the Y57K-R83Q double mutant was abolished completely (Fig. 4c,d), and the clean inward and outward currents seen at pH_o_ 5 and 10 were totally passive while the conductance was quite small (Supplementary Fig. 3a,c).

**Figure 5:**
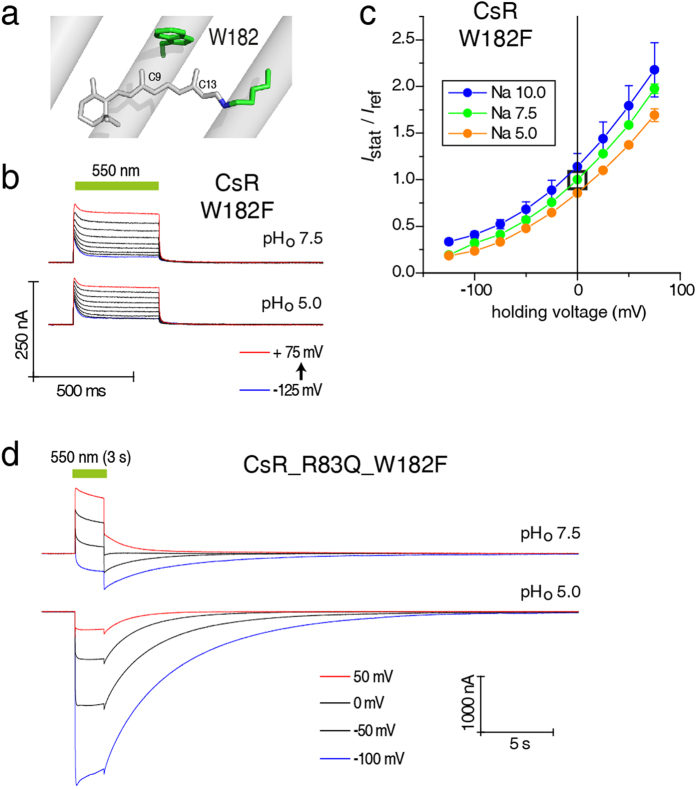
Inward-directed photocurrents produced by CsR-R83Q are channel-like. (**a**) Position W182 is highly conserved in microbial rhodopsins and is located close to the methyl groups of the retinal. (**b**) Photocurrents of CsR-W182F are characterized by a large transient outward peak compared to the stationary currents. (**c**) *I*_Stat_(*E*) of stationary photocurrents of CsR-W182F are always directed outward but curved compared to CsR-WT (550 ± 25 nm, normalized to pH 7.5 and 0 mV, pH 10 [n = 4], pH 7.5 [n = 6], pH 5 [n = 5]). (**d**) Similar to CsR-R83Q, photocurrents of CsR-R83Q-W182F are inward-directed, especially at low pH_o_. Additionally, the currents show drastically increased decay times. Data were measured in oocytes and represent the mean ± SE.

To understand the role of the photocycle intermediate that passively conducts protons in R83Q, we combined this mutation with W182F. W182 is a highly conserved residue of the retinal-binding pocket located above the retinal C11 position and interacting with both 9-methyl and 13-methyl groups (Fig. 5a)^17^. The absorption of BR following mutation of W182 to F became more than 70 nm blue-shifted^29^, and the transition from the L to M intermediate was dramatically slower^30^. Surprisingly, CsR-W182F showed active outward pumping with half the amplitude, but even faster kinetics than WT with more distinct inactivation at negative voltages (Fig. 5b,c). Nevertheless, the CsR-R83Q-W182F double mutant exhibited both active pumping and passive ion conductance. Pumping predominated over passive conductance in the light, but the pump was weak and the current tended to revert at negative voltages. After light-off the current exhibited a biphasic decay in which the pump currents decayed fast and passive conductance decayed slowly. At pH_o_ 5, however, only passive inward conductance with large amplitudes and slow off-kinetics was observed (Fig. 5d). There is clearly a slow equilibrium between a pumping and a conducting CsR with the ratio of the two activities dependent on the membrane voltage, pH_o_, and duration of illumination. Thus, we hypothesize that the W182F mutants perform a fast and a slow photocycle. Fast cycling is visible as active pumping whereas slow cycling does not contribute to the pumping process but rather to passive currents in cases where the CsR has been made conductive.

Next, we mutated the extracellular E193 and E203 residues (seen in Fig. 1) (Supplementary Fig. 4a–c). The corresponding residues E194 and E204 have been considered in several publications as part of the extracellular proton release machinery of BR^31^,^32^,^33^,^34^,^35^ without drawing any conclusion about the relevance of these residues for proton pumping.

Our studies show that the two release group residues are relatively unimportant for pumping activity and pump force of CsR. In both single mutants CsR-E193Q and CsR-E203Q, as well as in the CsR-E193Q-E203Q double mutant, pump activity at pH_o_ 10 was almost completely retained while current amplitudes were slightly reduced at pH_o_ 7.5 and 5. This was especially evident at negative voltages. These data suggest that proton release was less efficient at high extracellular proton concentration but the influence is relatively minor.

### Comparison with Bacteriorhodopsin (BR)

We identified two residues, R83 and Y57, as critical elements for active proton pumping of CsR. To determine whether these residues also play major roles in other proton pumps, we examined the effect of similar mutations on BR proton pump activity. Our data demonstrate that pumping by the BR-R82Q and BR-Y57K mutants was severely disrupted albeit to a different extent from CsR. Both still pumped weakly with moderate outward-directed currents at pH_o_ 10 and 7.5; however, at pH_o_ 5, the currents were passive and inward (Supplementary Fig. 5a–c). These results show that, like CsR, R82 and Y57 are important residues for active pumping in BR, with the nature of substitutions determining the degree and size of passive conductance.

### Proton uptake pathway of CsR

As explicitly shown for BR, the cytoplasmic (CP) channel of a light-driven pump is responsible for reprotonation of the RSB via a transiently formed water chain^18^. In BR, D96 is the key residue of the CP channel and is considered as the primary proton donor for reprotonation of the RSB during the M to N transition^36^,^37^,^38^,^39^,^40^. In Green-Proteorhodopsin and *Gloeobacter* rhodopsin, this aspartate is replaced by a glutamate that accelerates the reprotonation step but at the cost of leakiness in the pump at high load, i.e. low pH_o_ and negative voltage^41^. Taking a cue from these earlier results, we investigated the effect of mutating the analogous position, D97, in CsR. The CsR-D97E mutant was functional with reduced photocurrent amplitudes without inward-directed leak currents at any load (Supplementary Fig. 4d). Because the pK of D96 in BR is tuned by hydrogen bridging to T46^18^,^42^, similar results were expected for CsR (Fig. 1a). In CsR-T46N, photocurrents were enlarged at negative voltages and the kinetics were accelerated, indicating that this variant is even more effective and powerful than WT (Supplementary Fig. 6a,b). Promotion of the driving force for T46N was also visible in combination with R83Q. For CsR-T46N-R83Q, the passive conductance at low pH was reduced compared to the single mutant R83Q and the reversal voltages were more negative between pH_o_ 5 and 10 (Supplementary Fig. 6c,d).

#### MD simulations

**Figure 6:**
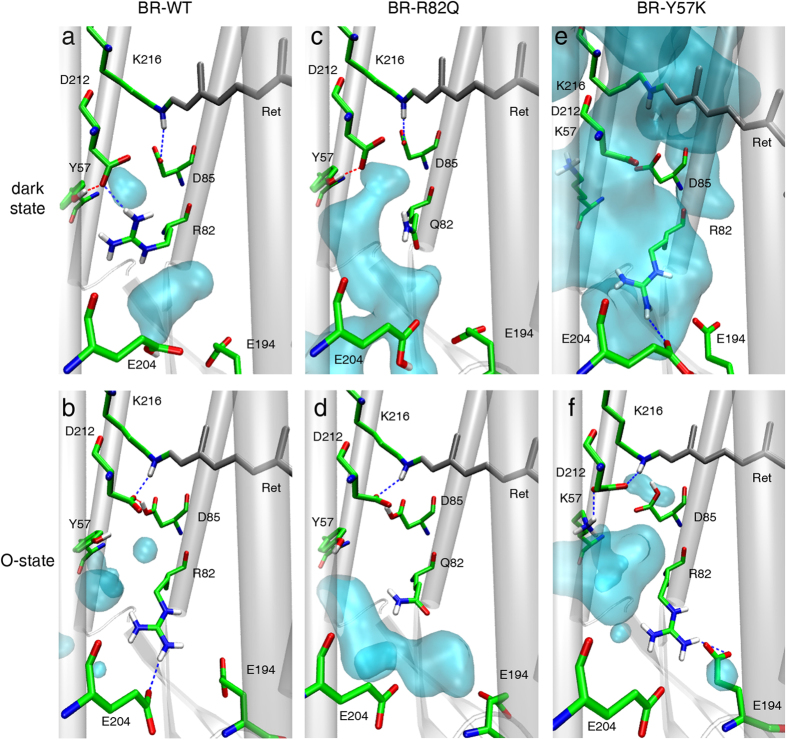
Molecular dynamics simulation and prediction of water molecule distribution of dark states and O-intermediates of BR. (**a**) The dark state of BR-WT with separated areas of mobile water. (**b**) In the O-state of BR-WT, the downward (extracellular side) movement of the guanidine side chain of R82 is depicted. The hydrogen bond connecting the guanidine group with Y57 and D212 was interrupted due to this downward movement. (**c**) Mutation of R82 by the smaller and neutral Q82 induced a large space with an extended water cluster. (**d**) Extended water cluster was still present in the O-state of R82Q, but direct accessibility to the primary proton donor D85 was interrupted. (**e**) Mutation of Y57 by the positively charged K57 promoted a salt bridge between D212 and K57. The dark state of K57Y was also characterized by an increased water cluster. (**f**) The O-state of Y57K lacks an interaction between R82 and E204. The huge cavity of mobile water molecules between K57, R82, D85, and D212 caused accessibility to the Schiff Base. Downward movement of the guanidine side chain of R82 was also observed.

To achieve a better understanding of the differences between WT pumps and R83/Y57 mutants, we calculated the structural differences between dark states and O-intermediates based on previously published BR structures^43^,^44^. During the BR dark-state, R82 is in direct contact with D212 (Fig. 6a), disrupting the water density between the active site and the H^+^-release residues E194 and E204. During the O-state, R82 faces outward to facilitate water invasion between R82 and D212, thereby forming a possible proton transfer pathway from D212 to E204 (Fig. 6b). The situation is quite different in the R82Q mutant where Q82 is oriented outward in both the dark- and O-states, allowing water to enter a channel between the active site and PRG. This structure forms a water wire in the dark-state and a proton transfer pathway that may involve Y57 in the O-state. The situation is more complicated for Y57K mutants. In the Y57K dark-state, K57 flips away from the counterions, leading to a very high water density in the active site, which is connected to the PRG. In the Y57K O-state model, however, K57 forms a salt bridge with D212, thereby neutralizing the charge of this counterion.

## Discussion

Throughout the years, numerous spectroscopists, crystallographers, and theoreticians have investigated the three elements of light-driven pumps: active site, proton release pathway, and Schiff base reprotonation. Unfortunately, in most cases except wild type BR, these studies did not evaluate the electrochemical gradients of the pumps *in vivo*. We demonstrated above quantitatively how the force, that a pump requires to overcome electrical or electrochemical gradients, depends on all three elements. Modification of any one element is sufficient to completely disrupt active pumping or convert the pump into a light-gated proton channel. However, the three elements have very different functions, as discussed below.

Many studies have investigated the role of the active site. Our photocurrent measurements of CsR-D86T are consistent with a model in which the primary proton acceptor is absent and photoexcitation causes release of the Schiff base (SB) proton to the intracellular side of the medium as previously proposed^45^. We expect that in this case deprotonation occurs late during the photocycle when the water chain between the SB and internal bulk phase is established. The fact that stationary outward currents are observed for CsR-D86T with a deprotonated dark-state chromophore at high pH_o_ (Fig. 2d–f) may imply that, after photoisomerization of deprotonated all-*trans* retinal into *13-cis*, the RSB retrieves a proton from the intracellular compartment and releases it to the extracellular side during thermal reisomerization.

The fact that D211N also promotes passive currents even though the RSB remains protonated in the dark-state favors a model in which disturbance of the proton network during one of the late photocycle intermediates (N or O) is important and not the protonation state of the counter ion. Moreover, the passive currents of CsR-D86T and CsR-D211N are small (Supplementary Fig. 2c), suggesting that these mutants retained a 1:1 coupling between photoisomerization and proton translocation and do not promote a channel-like conductance. The reduced currents observed with CsR-D86T compared to CsR-D211N can be explained by the lower quantum efficiency of deprotonated RSB chromophores^46^.

The effects of CsR-W182F (Fig. 5) were unexpected. The homologous mutants in BR and Sensory Rhodopsin II (SRII) have been described as mutants with slow photocycle and a particularly slow L to M and M to N conversion^29^,^47^,^48^ which seemed at a first glance inconsistent with our results of CsR-W182F that produced significant pump currents. However, CsR-R83Q-W182F revealed a dual function for this W182F mutation with fast pump currents and slow passive conductance, suggesting that the two photocycles may represent two populations that operate in an independent manner. Weidlich, *et al.* proposed that retinal isomerization is distorted in BR-W182F^47^. Consistent with this argument, it is conceivable that two photocycles, one with 13*-cis*,15-*anti* M-state and the other with *at,*15-*syn*, coexist as photoproducts from *at,*15*-anti* (*trans*-cycle) and 13-cis,15-syn (*cis*-cycle) dark-states, respectively. Such a phenomenon has been reported for dark-adapted BR (1:1)^49^ and channelrhodopsin (0.7:0.3)^50^.

**Figure 7:**
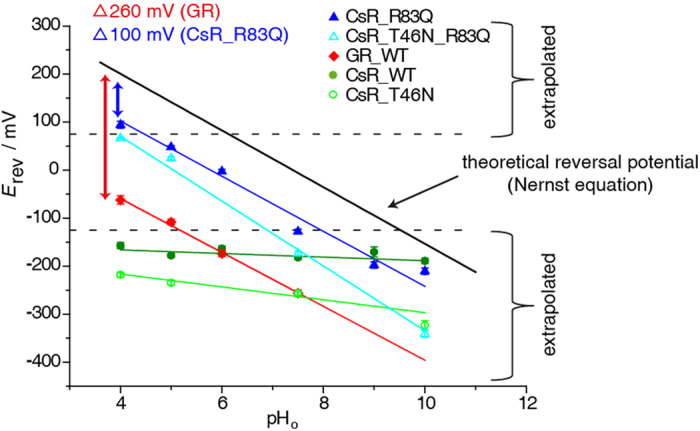
Reversal voltages of Δp- and ΔΨ-type light-driven proton pumps. Reversal voltage (*E*_rev_) of *Gloeobacter* rhodopsin (GR), CsR-WT, CsR-T46N, CsR-R83Q, and CsR-R83Q-T46N are plotted against the extracellular pH_o_. The black line represents the theoretical expected *E*_rev_ of free proton flow calculated by the Nernst equation based on an intracellular pH_i_ of 7.4. Reversal potentials below this line indicate proton pump activity. Values from −125 mV up to +75 mV were determined directly. Other values are determined by extrapolation. CsR-WT (550 ± 25 nm, pH 10 [n = 8], pH 9 [n = 3], pH 7.5 [n = 26], pH 6 [n = 5], pH 5 [n = 8], pH 4 [n = 6]) exhibited high resistance against low pH_o_ whereas GR (400–600 nm, pH 7.5 [n = 23], pH 6 [n = 6], pH 5 [n = 9], pH 4 [n = 10]) was shifted −260 mV in parallel to the calculated potentials. Mutation of CsR-R83 (550 ± 25 nm, pH 10 [n = 7], pH 9 [n = 4], pH 7.5 [n = 11], pH 6 [n = 4], pH 5 [n = 6], pH 4 [n = 4]) into Glu converts *Coccomyxa* rhodopsin into a *Gloeobacter*-like proton pump in relation to the *E*_rev_. CsR-T46N (550 ± 25 nm, pH 10 [n = 4], pH 7.5 [n = 8], pH 5 [n = 4], pH 4 [n = 4]). CsR-T46N-R83Q (550 ± 25 nm, pH 10 [n = 4], pH 7.5 [n = 5], pH 5 [n = 5], pH 4 [n = 2]). Data were measured in oocytes and represent the mean ± SE.

The extracellular proton release pathway has also been the subject of intense research. We calculated the reversal voltages *E*_rev_ for WT and mutant CsRs, as well as compared the values with the theoretical expected *E*_rev_ for passive proton flow according to the Nernst equation (Fig. 7 and Supplementary Figs 2a and 3c). In contrast to Nernst, *E*_rev_ for WT changes only weakly with the pH_o_ from −190 mV at pH_o_ 10 to −150 mV at pH_o_ 4, similar to BR. Considering the power of the CsR pump relative to the proton motive force (Δp), it increases from −40 mV at pH 10 up to −360 mV at pH 4, revealing that, in principle, the pump is very strong. However, at higher pH, this power cannot be exploited for reasons that are unknown (ΔΨ-pump). This is unlikely a thermodynamic limitation but rather due to modification of the hydrogen bonding network or protonation stages of essential residues that are responsible for this limitation. But, it was totally unexpected that modification of the pK of CsR-D97 by replacement of its hydrogen bond partner T46 with N (Fig. 7 and Supplementary Fig. 6) the power of the Δ Ψ−pump increased over the CsR-WT pump by almost −70 mV.

In contrast, the R83Q mutant was able to pump against a −100 mV electrochemical gradient (proton motive force Δp) over the whole pH_o_ range from 4 to 10 (Δp-pump). At any condition exceeding this Δp value, the current switches to passive inward transport (Fig. 7). Thus, the characteristics of this pump are completely different from CsR-WT but similar to *Gloeobacter* rhodopsin, which also possesses a strong dependence on pH at a pumping power of −260 mV at all pH values typical for a Δp-pump (Fig. 7).

The fact that proteorhodopsins do not contain E194 and E204 in the PRG and in parallel exhibit a higher pK of the D85 counterion prompted researchers to conclude that these two glutamates are the parameters that allow ΔΨ-pumps including BR to remain insensitive to the external pH^51^. However, the small reduction of currents produced by the PRG-deficient mutant at pH_o_ 10, and the equal pumping observed at pH_o_ 5 and 7.5, also questions the importance of these two residues for pH-insensitivity. In contrary, studies in BR demonstrate that replacement of BR-R82 with Q increases the pK of the SB D85 counterion from 2.5 to 7.2 at 150 mM NaCl^26^,^52^. FTIR measurements of BR-WT and R82A led to the suggestion that R82 is deprotonated during M-formation and is reprotonated via D85 late in the photocycle during O to BR_dark_ transition^53^. However, this hypothesis is not generally accepted^15^. MD simulations, including those presented here, favor formation of a proton-conducting water chain that is formed during N to O transition (Fig. 6b). Further, our MD simulations support the conclusion that in the CsR-R83Q and Y57K mutants such a water chain between RSB and the extracellular bulk phase already exists in darkness and probably during the entire photocycle. Thus, when the intracellular water chain is established during the M to N transition, the mutants become capable of conducting protons.

## Methods

### Cloning, heterologous expression, and mutagenesis of CsR

A human codon-adapted COCSUDRAFT_42526 gene (Gene ID: 17040130, protein accession number I0YUS5) was synthesized by Geneart (Germany) and cloned into the pGEMHE vector. Site-directed mutagenesis was performed using the QuikChange Kit (Agilent Technologies, Santa Clara, CA) according to the manufacturer’s instructions.

### Electrophysiology with Xenopus laevis oocytes

Preparation of *Xenopus laevis* oocytes^54^ and expression of microbial rhodopsins were performed as described previously^41^. Briefly, 25–35 ng capped-*mRNA* were injected per oocyte and incubated at 18 °C in ORI solution supplemented with 5 μM all-*trans* retinal (Sigma, St. Louis, MO). Oocytes were measured 4–5 days after injection.

A two-electrode voltage clamp was performed with an amplifier, Turbo Tec-10×, without transient compensation (NPI Electronic GmbH, Tamm, Germany). Continuous light was provided by a 75 W Xenon lamp, XBO75/2 (Karl Zeiss GmbH, Jena, Germany). The light passed through broadband filters (K55 filter for 550 nm light, K40 filter for 400 nm light, Optics Balzers AG, Lichtenstein) with an intensity of 12 mW/cm^2^. For action spectra measurement, polychrome II (Till photonics, Munich, Germany) was used as a light source and currents were normalized to the corresponding photon flux at each wavelength. The resistances of microelectrodes were kept between 0.4 and 1.2 MΩ. Data acquisition and light triggering were controlled with pCLAMP 9.0 software via the DigiData 1322A interface (Molecular Devices, Sunnyvale, CA, USA). Currents were recorded at 10 kHz and filtered to 1 kHz by built-in circuits. Shown current traces were additionally filtered to 500 Hz (Gauss-lowpass cutoff filter using pCLAMP). The composition of standard extracellular buffer solutions was as follows: 100 mM NaCl, 1 mM MgCl_2_, 0.1 mM CaCl_2_, 5 mM MOPS (pH_o_ 7.5, adjusted with 1 M NMG), 5 mM glycine (pH_o_ 10.0, adjusted with 1 M NMG), and 5 mM citric acid/Na-citrate (pH_o_ 5.0, adjusted with citric acid/Na-citrate). Buffers with different compositions than these are described in the appropriate section.

### Culturing, confocal microscopy, and patch-clamp experiments on HEK-293 cells

Culturing, confocal microscopy, and patch-clamp experiments on HEK-293 cells were performed as described previously^41^. Cells were supplemented with 1 μM all-*trans* retinal (Sigma) and analyzed 24–30 hours post-transfection.

### Molecular dynamics (MD) simulation

To gain insight into the structural and dynamic properties of BR, we performed 50 ns molecular dynamics (MD) simulations. The x-ray structure of BR at 1.55 Å resolution (PDB ID: 1C3W)^43^ was used as the starting point for dark states. Since a crystal structure for the wild type O-state has yet to be published, we constructed the O-state structure based on its main characteristics, namely all-*trans* retinal, neutral D85, and deprotonated proton release group (E194, E204). The MMTSB tool set^55^ was used to simulate the point mutations R82Q and Y57K. The protein was embedded into a 1-palmitoyl-2-oleoylphosphatidylcholine bilayer and explicit TP3P^56^ water molecules were used as solvent. The complex was neutralized with chloride anions and was described with the Charmm36 classical force field^57^. The production simulations employed a time step of 2 fs, and the lincs algorithm was applied to keep all covalent bonds constrained at their respective reference distances. Regarding the treatment of non-bonded interactions, a cut-off of 1.2 nm was used for the van der Waals interactions, while the particle-mesh Ewald implementation^58^ was used to evaluate the point-charge electrostatics. All MD simulations are performed using Gromacs version 4.6.1^59^.

### Computation of water distribution and complex structure

The VolMap plug-in implemented in VMD 1.9.1 was used to compute the water distribution as the mass weighted atomic densities were averaged over all frames over a 50 ns trajectory. The 3D grid resolution was set at 1 Å and the atom size value was set to 1 and the default cutoffs for the water densities were used. The resulting OpenDX voxels of the average water densities were then analyzed using dxTuber, a grid-based cavity detection method^60^, with default settings to derive the PDB format output encoded as formal b-factors. The MD trajectories were aligned to the starting structure of the production simulations based on Cα atoms prior to VolMapping to eliminate protein drift in the simulation box. The most likely structures^61^ of the complexes were derived utilizing the gromacs tool g_mlstr, in which the position of the side chain atoms is determined by dihedral angle analysis while the backbone atoms are averaged.

## Additional Information

**How to cite this article**: Vogt, A. *et al.* Conversion of a light-driven proton pump into a light-gated ion channel. *Sci. Rep.* **5**, 16450; doi: 10.1038/srep16450 (2015).

## Supplementary Material

Supplementary Information
