# Supplementary material for: Conversion of a light-driven proton pump into a light-gated ion channel
Source: Sci Rep. 2015 Nov 24;5:16450. doi: 10.1038/srep16450 (PMC4657025; doi:10.1038/srep16450)
Supplement: Supplementary Information [file srep16450-s1.pdf]

Supplementary Information

Conversion of a light-driven proton pump into a light-gated ion channel

A.Vogt[1], Y. Guo[2], S. P. Tsunoda[1,#], S. Kateriya[3], M. Elstner[2], and P. Hegemann[1]

[1]Institute of Biology, Experimental Biophysics, Humboldt-Universität zu Berlin,

10115 Berlin, Germany

[2]Institute of Physical Chemistry, Karlsruhe Institute of Technology,
76131 Karlsruhe, Germany

[3]Department of Biochemistry, University of Delhi South Campus, New Delhi, India

# current affiliation: Department of Frontier Materials, Nagoya Institute of Technology,
Showa-ku, Nagoya 466-8555, Japan

To whom correspondence may be addressed: Peter Hegemann, Experimental Biophysics, Institute of Biology, Humboldt-Universität zu Berlin, Invalidenstr. 42, 10115 Berlin, Germany, Tel.: +49 30 2093-8681; Fax: +49 30 2093-8520; E-mail: hegemape@rz.hu-berlin.de

```
CsR          -------------------MAVHQIGEGGLVMYWVTFG-------------LMAFSALAFA 29
BR           ------------------QAQIT----GRPEWIWLALGTA-----------LMGLGTLYFL 28
Arch-3       ------------MDPIALQAGYDLLGDGRPETLWLGIGTL-----------LMLIGTFYFL 38
GR           -MLMTVFSSAPELALLGSTFAQVDPSNLSVSDSLTYGQFNLVYNAFSFAIAAMFASALFF 59
PR           MKLLLILGSVIALPTFAAGGGDLDASDYTGVSFWLVT-------------AALLASTVFF 47
HR           -MSITSVPGVVDAGVLGAQSAAAVRENALLSSSLWVNVA-----------LAGIAILVFV 48
                                       .                          :.   *

CsR          VMTFTRP-LNKRSHGYITLAIVTIAAIAYYAMAASGGKALVSN-----PDGNLRDIYYAR 83
BR           VKGMGVSDPDAKKFYAITTLVPAIAFTMYLSMLLGYGLTMVP------FGGEQNPIYWAR 82
Arch-3       VRGWGVTDKDAREYYAVTILVPGIASAAYLSMFFGIGLTEVT------VGGEMLDIYYAR 92
GR           FSAQALVGQRYRLALLVSAIVVSIAGYHYFRIFNSWDAAYVLENGVYSLTSEKFNDAY-R 118
PR           FVERDRVSAKWKTSLTVSGLVTGIAFWHYMYMRGVWIETGDSP-------------TVFR 94
HR           YMGRTIRPGRPRLIWGATLMIPLVSISSYLGLLSGLTVGMIEMPAGHALAGEMVRSQWGR 108
                        :     :  :  ::   *  :                           *

CsR          YIDWFFTTPLLLLD----IILLTGIPIGVTLWIVLADVAMIMLGLFGALS--TNSYRWGY 137
BR           YADWLFTTPLLLLD----LALLVDADQGTILALVGADGIMIGTGLVGALTK-VYSYRFVW 137
Arch-3       YADWLFTTPLLLLD----LALLAKVDRVTIGTLVGVDALMIVTGLIGALSH-TAIARYSW 147
GR           YVDWLLTVPLLLVETVAVLTLPAKEARPLLIKLTVASVLMIATGYPGEISD-DITTRIIW 177
PR           YIDWLLTVPLLICEFYLILAAATNVAGSLFKKLLVGSLVMLVFGYMGEAG---IMAAWPA 151
HR           YLTWALSTPMILLA----LGLLADVDLGSLFTVIAADIGMCVTGLAAAMTTSALLFRWAF 164
             *  *::.*:::      :   .         :   .  *   *  .

CsR          YGVSCAFFFVVLWGLFFP-GAKGARARGGQVPGLYFGLAGYLALLWFGYPIVWGLAE-GS 195
BR           WAISTAAMLYILYVLFFG-FTSKAESMRPEVASTFKVLRNVTVVLWSAYPVVWLIGSEGA 196
Arch-3       WLFSTICMIVVLYFLATS-LRSAAKERGPEVASTFNTLTALVLVLWTAYPILWIIGTEGA 206
GR           GTVSTIPFAYILYVLWVE-LSRSLVRQPAAVQTLVRNMRWLLLLSWGVYPIAYLLPMLGV 236
PR           FIIGCLAWVYMIYELWAGEGKSACNTASPAVQSAYNTMMYIIIFGWAIYPVGYFTGYLMG 211
HR           YAISCAFFVVVLSALVTD-WAASASSAG--TAEIFDTLRVLTVVLWLGYPIVWAVGVEGL 221
               ..      ::  *               .      :     . *  **: :

CsR          DYIS-VTAEAASYAGLDIAAKVVFGWAVMLSHPLIARNQTDGSLLINSTNDPFVASTTHI 254
BR           GIVP-LNIETLLFMVLDVSAKVGFGLILLRSRAIFG------------------------ 231
Arch-3       GVVG-LGIETLLFMVLDVTAKVGFGFILLRSRAILG------------------------ 241
GR           SGTSAAVGVQVGYTIADVLAKPVFGLLVFAIALVKT------------------------ 272
PR           DGGS-ALNLNLIYNLADFVNKILFGLIIWNVAVKES------------------------ 246
HR           ALVQSVGVTSWAYSVLDVFAKYVFAFILLRWVANNE------------------------ 257
                         :   *.  *  *.  :

CsR          PERQGGIFGGLMGKKRGAGTPLATNEGVPRKAAPTAATTTAGNPATAAEV------ 304
BR           --------------------------------EAEAPEPSAGDGAAATSD------ 249
Arch-3       --------------------------------DTEAPEPSAGADVSAAD------- 258
GR           ------------------------------KADQESSEPHAAIGAAANKSGGSLIS 298
PR           ----------------------------------------SNA------------- 249
HR           --------------------------------RTVAVAGQTLGTMSSDD------- 274
                        :
```

Supplementary Figure 1: Protein sequence alignment of selected microbial pumps. Alignment was performed by using ClustalW2 (version CLUSTAL 2.1, provided by EMBL-EPI, Europe). Mutated positions of CsR are highlighted in black frames. Coccomyxa Rhodopsin (abbreviated as CsR, protein accession number I0YUS5), Bacteriorhodopsin (BR, P02945), Archaerhodopsin-3 (Arch-3, P96787), Gloeobacter Rhodopsin (GR, Q7NP59), Green-light absorbing proteorhodopsin (PR, Q9F7P4), Halorhodopsin (HR, P16102)

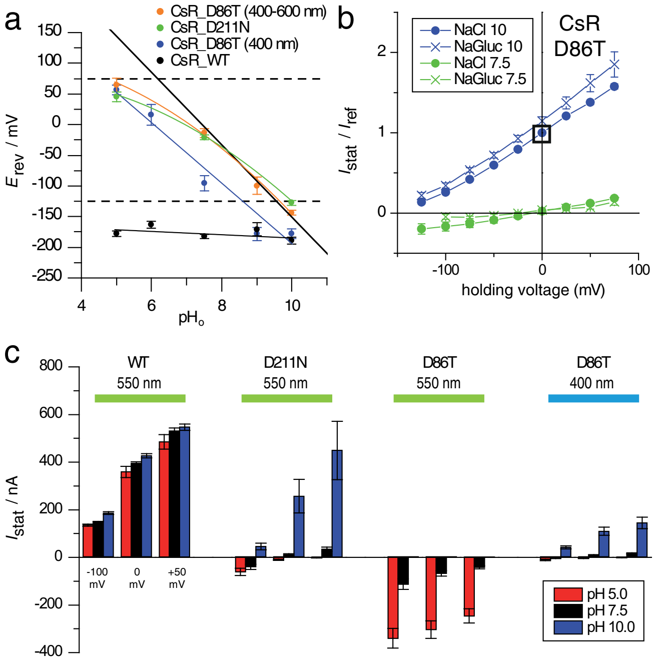

Supplementary Figure 2: Mutants of the counterion complex. (**a**) Reversal potentials (E_{rev}) of stationary photocurrents of CsR-WT, D86T (illuminated with 400 nm or 400-600nm), and D211N. The black line represents the theoretical expected E_{rev} of free proton flow calculated by the Nernst equation based on an intracellular pH_i of 7.4. Reversal potentials below this line indicate proton pump activity. Values from -125 mV up to + 75 mV were determined directly. Other values are determined by extrapolation. CsR-WT (550 ± 25 nm, taken from Fig. 7). CsR-D86T (400 ± 25 nm, n=4-14), CsR-D86T (400-600 nm, n=5-25), CsR-D211N (550 ± 25 nm, n=8-9). (**b**) $I_{Stat}(E)$ of stationary photocurrents of CsR-D86T to test the potential contribution by chloride. No appreciable difference was observed following exchange of 100 mM NaCl with 100 mM sodium gluconate (NaGluc) (400-600 nm, normalized to pH 7.5 and 0 mV with 100 mM NaCl, NaCl pH 10 [n=22], NaGluc pH 10 [n=10], NaCl pH 7.5 [n=11], NaGluc pH 7.5 [n=5]). (**c**) Absolute photocurrents amplitudes at different pH_o and holding potentials. In general, amplitudes of CsR-D86T were weak compared to WT and other mutants. CsR-WT (550 ± 25 nm, n=7-25), inward peak currents of CsR-D86T (550 ± 25 nm, pH 7.5 [n=10], pH 5 [n=3]), stationary current of CsR-D86T (400 ± 25 nm, n=7-14), CsR-D211N (550 ± 25 nm, n=7-9). Data were measured in oocytes and represent the mean ± SE.

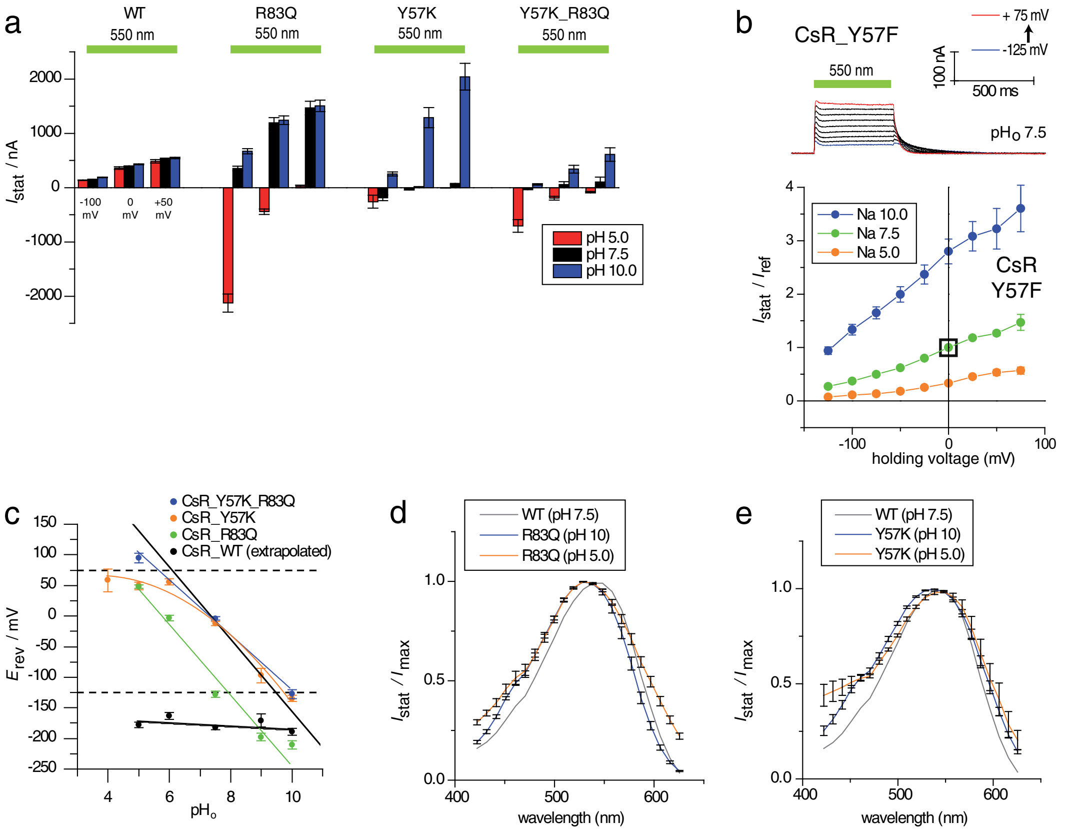

Supplementary Figure 3: Current traces and $I_{Stat}(E)$ relationship of individual and combined R83Q and Y57K mutants. In all experiments cells were illuminated with 550 ± 25nm. (**a**) Absolute photocurrent amplitudes at different pH_o and holding potentials. CsR-WT (n=7-25), CsR-R83Q (n=6-7), CsR-Y57K (n=6-12), and CsR-Y57K-R83Q (n=8-11). (**b**) Photocurrents of CsR-Y57F at pH_o 7.5 and $I_{Stat}(E)$ show that mutation at position Y57 does not always lead to inward-directed photocurrents (550 ± 25 nm, normalized to pH 7.5 and 0 mV, pH 10 [n=9], pH 7.5 [n=13], pH 5 [n=8]). (**c**) Reversal potentials (E_{rev}) of stationary photocurrents. The black line represents the theoretical expected E_{rev} of free proton flow calculated by the Nernst equation based on an intracellular pH_i of 7.4. Reversal potentials below this line indicate proton pump activity. Values from -125 mV up to + 75 mV were determined directly. Other values are determined by extrapolation. CsR-WT (taken from Fig. 7), CsR-Y57K (n=2-12), CsR-R83Q (n=4-11), and CsR-Y57K-R83Q (n=3-10). (**d-e**) Action spectra of CsR-R83Q and CsR-Y57K measured at pH 10 and pH 5.0 with supplementation of 5 µM all-*trans* retinal. Currents were recorded at 0 mV and normalized to the maximal stationary currents. (WT at pH 7.5 [n=16]; R83Q at pH 10 [n=5], R83Q at pH 5.0 [n=4], Y57K at pH 10 [n=4], Y57K at pH 5.0 [n=6]). Data were measured in oocytes and represent the mean ± SE.

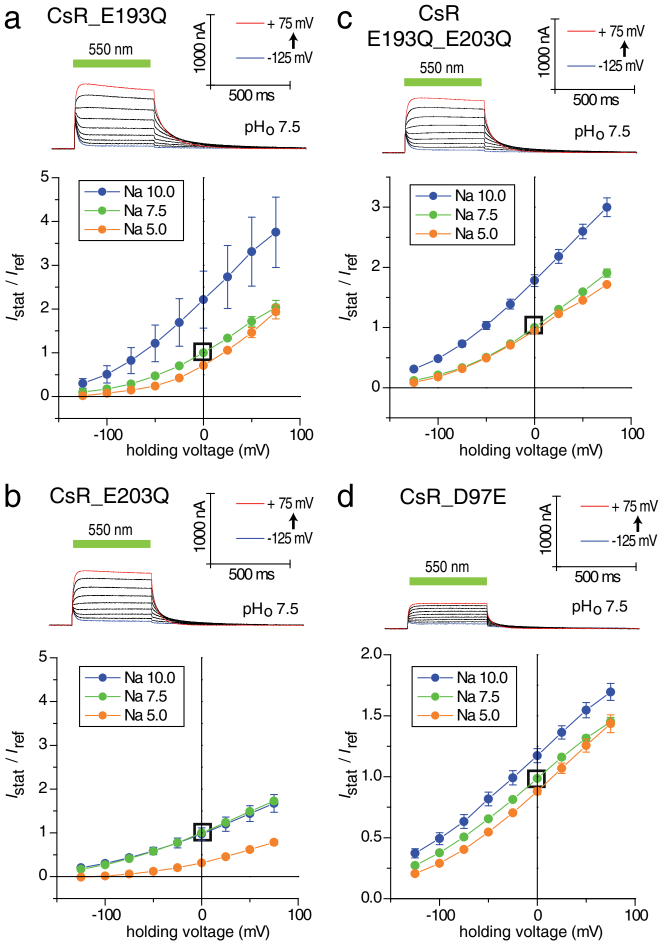

Supplementary Figure 4: Mutants of the proton release complex and the primary proton donor D97. All experiments were illuminated with 550 ± 25 nm and normalized to pH 7.5 and 0 mV. (**a**) Photocurrents of CsR-E193Q at pH_o 7.5 and $I_{Stat}(E)$ (pH 10 [n=8], pH 7.5 [n=11], pH 5 [n=4]). (**b**) Photocurrents of CsR-E203Q at pH_o 7.5 and $I_{Stat}(E)$ (pH 10 [n=5], pH 7.5 [n=7], pH 5 [n=3]). (**c**) Photocurrents of CsR-E193Q-E203Q at pH_o 7.5 and $I_{Stat}(E)$ (pH 10 [n=5], pH 7.5 [n=7], pH 5 [n=4]). (**d**) Photocurrents of CsR-D97E at pH_o 7.5 and $I_{Stat}(E)$ showed decelerated kinetics without inward currents as seen in proteorhodopsin-like pumps where glutamate is conserved at this position (pH 10 [n=5], pH 7.5 [n=9], pH 5 [n=4]). Data were measured in oocytes and represent the mean ± SE.

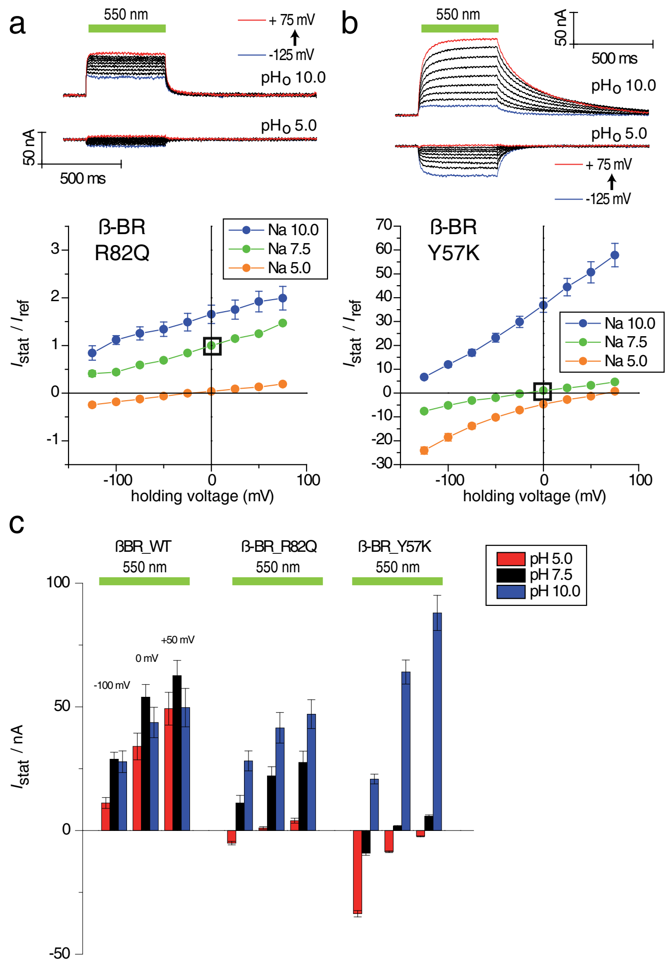

Supplementary Figure 5: BR mutants βBR-R82Q and βBR-Y57K show inward- and outward-directed photocurrents. All experiments were illuminated with 550 ± 25nm and normalized to pH 7.5 and 0 mV. (**a**) Photocurrents of βBR-R82Q at pH_o 10 and 5. $I_{Stat}(E)$ is shown below (pH 10 [n=5], pH 7.5 [n=8], pH 5 [n=4]). (**b**) Photocurrents of βBR-Y57K at pH_o 10 and 5. $I_{Stat}(E)$ is shown below (pH 10 [n=6], pH 7.5 [n=6], pH 5 [n=5]). (**c**) Absolute photocurrents amplitudes at different pH_o and holding potentials. βBR-R82Q and βBR-Y57K inward- and outward-directed photocurrents were weaker compared to the corresponding mutants of CsR. (βBR-WT n=8-17, βBR-R82Q n=4-8, and βBR-Y57K n=5-6). Data were measured in oocytes and represent the mean ± SE.

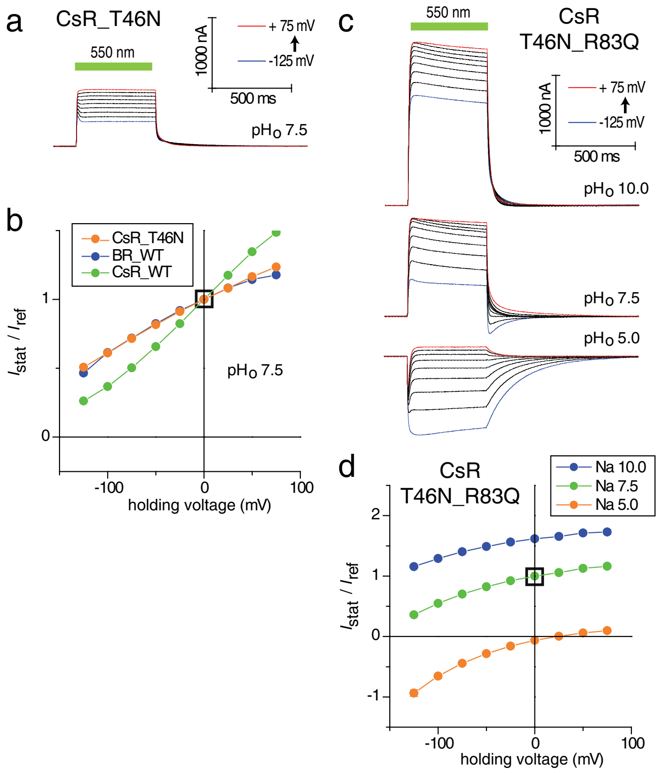

Supplementary Figure 6: Function of the CsR proton uptake channel. All experiments were illuminated with 550 ± 25nm and normalized to pH 7.5 and 0 mV. (**a**) Photocurrent traces of CsR-T46N at pH_o 7.5. (**b**) $I_{Stat}(E)$ of CsR-WT compared to CsR-T46N and BR-WT at pH 7.5. The slope of the $I_{Stat}(E)$ of CsR-WT is steeper (i.e., more sensitive to the holding potential) compared to BR-WT. This is a basic difference between CsR-WT and BR-WT. CsR-T46N produced the same slope as BR-WT, indicating that this difference is caused by the architecture or dynamics of the proton uptake channel. (**c**) Photocurrents of CsR-T46N-R83Q at different pH_o. (**d**) $I_{Stat}(E)$ are shown (pH 10 [n=4], pH 7.5 [n=5], pH 5 [n=5]). Inward-directed photocurrents caused by R83Q were still present at low pH. The E_{rev} was shifted slightly by the quantity caused by the single mutation T46N, indicating that both single mutants act independent of each other and that their effects are additive. Data were measured in oocytes and represent the mean ± SE.
